# Sustainable Agriculture Development in Northwest China Under the Impacts of Global Climate Change

**DOI:** 10.3389/fnut.2021.706552

**Published:** 2021-11-05

**Authors:** Dachuan Liu, Yan Li, Pengfei Wang, Huaqi Zhong, Pu Wang

**Affiliations:** ^1^Institutes of Science and Development, Chinese Academy of Sciences (CAS), Beijing, China; ^2^Yulin Agricultural Publicity and Information Centre, Yulin, China; ^3^National Engineering Laboratory for Lake Pollution Control and Ecological Restoration, Chinese Research Academy of Environmental Sciences, Beijing, China; ^4^State Environment Protection Key Laboratory for Lake Pollution Control, Chinese Research Academy of Environmental Sciences, Beijing, China; ^5^South China Institute of Environmental Sciences, Ministry of Ecology and Environment, Guangzhou, China; ^6^School of Public Policy and Management, University of Chinese Academy of Sciences (UCAS), Beijing, China

**Keywords:** sustainable agriculture, climate change adaptation, climate disaster, food security, vulnerability, resilience

## Abstract

Northwest China has one of the most vulnerable agricultural systems in the context of global climate change. We argue that sustainable agriculture development in this region requires a systematic approach toward climate change adaptation, and propose a schematic framework for strategic thinking. We first briefly review the impacts of climate change on various agricultural environmental factors, including light, temperature, water, and atmosphere, and explores the effects of climate change on agricultural practices, such as disaster response, pests and weeds control, fertilizer application, and species selection. The study shows that climate change has increased extreme climate disasters such as drought and heat waves, and has expanded the scope and severity of pests and weeds, which in turn requires a series of changes in farming practices. These effects have profound impacts on farmland management, as well as the sustainability of the agricultural system. Based on the findings, the authors argue that the key adaptation strategies should include: (1) optimizing the geographic distribution of agriculture, (2) cultivating new crop varieties that can better adapt to the changing environment, (3) adjusting cropping timing and structure, (4) developing water-saving irrigation systems, (5) improving capacities of disaster prevention and mitigation at both household and government levels, and (6) strengthening the sciences, technology, and human resources to mitigate the adverse effects of climate change.

## Introduction

**Figure 1:**
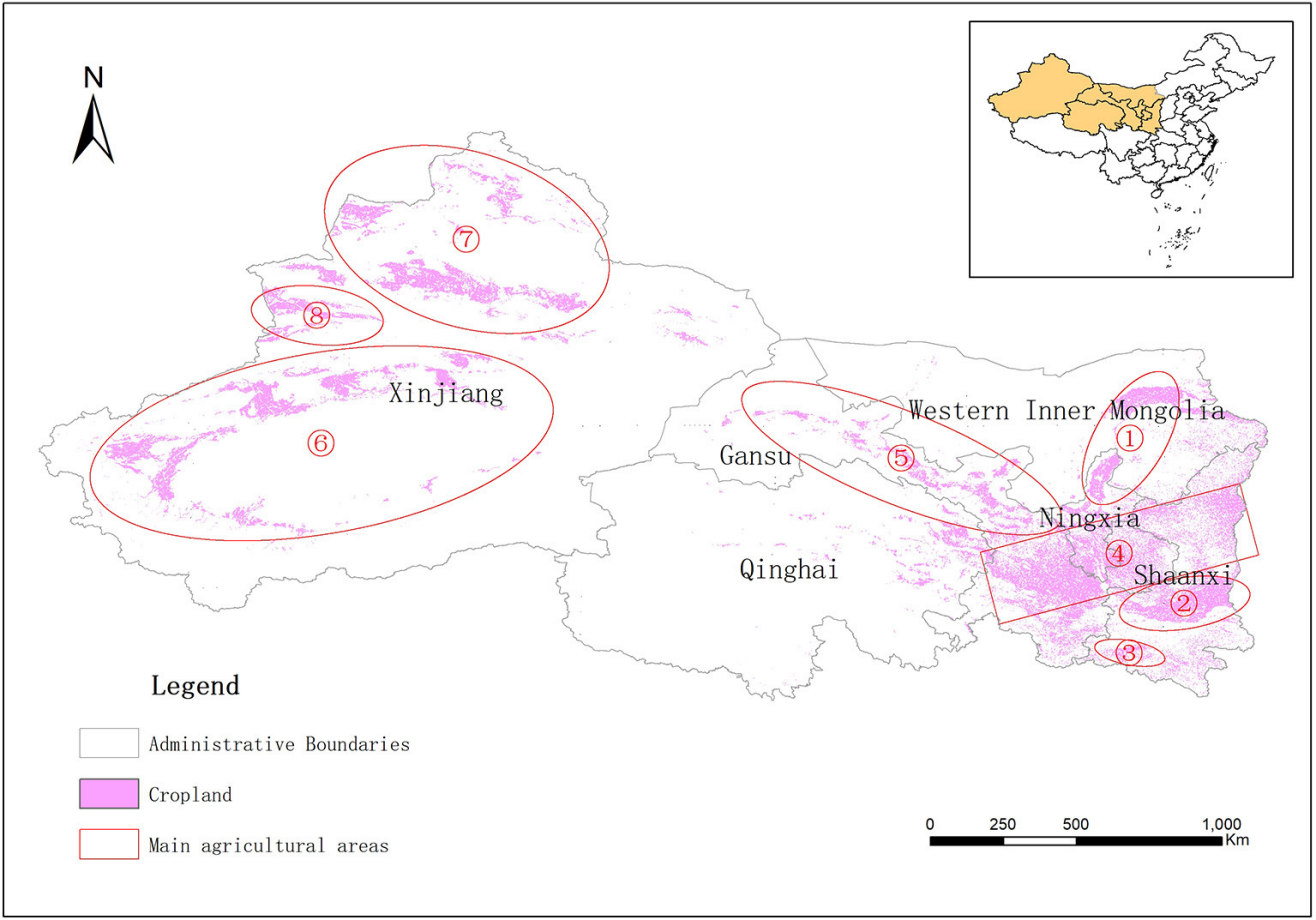
Main agricultural areas in Northwest China. ① Hetao Plain, arid area; ② Guanzhong Plain, semi-humid area; ③ Hanzhong Plain, wet area; ④ Loess Plateau, Semi-arid area; ⑤ Hexi Corridor, arid area; ⑥ Tarim Basin, arid area; ⑦ Junggar Basin, arid area; ⑧ Yili Valley, semi-arid area. Data obtained from the GlobeLand30 dataset (7).

Agriculture is one of the most vulnerable economic sectors that are increasingly affected by global climate change (1, 2), particularly for populous developing countries such as China (3–5). Northwest China includes five provinces, namely Xinjiang, Qinghai, Gansu, Ningxia, and Shaanxi (6); due to their similar geographic and climatic conditions to Northwest China, the four prefectures in western Inner Mongolia (Alashan, Ordos, Wuhai, and Bayannur) are also included for our discussion (Figure 1). The agriculture sector in Northwest China is particularly vulnerable to climate change (8) for two reasons. First, the topography of this region is dominated by plateaus and mountains, with large areas of deserts and Gobi and scattered agricultural plains due to scarcity of water resources, which is significantly different from main agricultural regions in Northeast and Central China (9, 10). Second, economic development in Northwest China is lagging behind the rest of the country, and the irrigation and other agricultural infrastructure systems are underdeveloped, so the capacity to adapt to climate change is considerably weak (11).

However, the impacts of climate change on agriculture in Northwest China has not received adequate attention in national and regional studies (12–14); particularly, a long-term, systematic thinking on adaption of agricultural system to climate change is lacking (15). We argue that climate change needs to be considered as one of the most important variables in agriculture policy making and planning in Northwest China. Comprehensive adaptation strategies, including adjusting the geographic scope of agricultural activities, crop variety selection, farmland management measures, and climate disaster responses, need to be carefully studied.

To support our perspective, we reviewed representative studies related to sustainable development of agriculture in Northwest China. Academic publications are selected based on the following criteria: (1) general studies related to sustainable agriculture in Northwest China, (2) quantitative studies analyzing the changing trends of light, heat, water, and atmosphere, providing basic data to support an overview of the changes in the environmental factors, (3) studies focusing on agricultural practices to provide evidence to assess the impacts of climate change on crop phenology, farming practices, and extreme climate disasters response, (4) policy studies that help make policy recommendations for sustainable development of agriculture.

Based on the review of representative literature, the aim of this perspective paper is to propose a framework for systematic thinking on sustainable agricultural development in Northwest China in the context of climate change. To this end, we establish a logic chain of analysis: changing trends in agricultural environmental elements—impacts on agricultural practices—corresponding adaptation strategies. First, climate change has directly or indirectly affected the key agricultural environmental elements, including light, heat, water, and atmosphere. These changes in agricultural environmental elements can affect crop phenology, pests and weeds, extreme climate disasters, and further impact agricultural practices, both favorably and unfavorably. To adapt to these impacts, a series of adaptation measures should be taken, such as optimizing geographic distribution, adjusting crop composition, breeding new varieties for climate adaptation, and enhancing disaster response capacity. We review the impacts of climate change on agricultural environmental elements and farming practices in section Impacts of Climate Change on Environmental Factors and Agricultural Practices in Northwest China, propose adaptation strategies in section Strategies to Adapt to Climate Change, and discuss the key implications of our study in section Discussion.

## Impacts of Climate Change on Environmental Factors and Agricultural Practices in Northwest China

### Impacts on Light, Heat, Water, and Atmosphere

Northwest China has abundant light resources due to the dry climate and high altitudes (16, 17). The total solar radiation ranges between 4,300 and 7,000 MJ/(m^2^·a) (17) and the average annual sunshine hours range between 1,500 and 3,400 h (16). As a result of climate change, clouds, aerosols, and humidity have increased in this region, which have caused decrease in solar radiation and sunshine hours in most areas (16–19). Chen, Xing (17) showed that the total solar radiation in Northwest China decreased at a rate of −92.07 MJ/(m^2^·10a) from 1961 to 2003; Xu, Yang (18) showed that the overall annual sunshine hours decreased from 1961 to 2007 at a rate of −24h/10a; similar studies also concluded that the sunshine hours decreased in most parts of the region (16, 19). Reduction in solar radiation and sunshine hours can lead to lower crop yields, though the extent is limited.

Due to climate change, agricultural heat resources in Northwest China show an increasing trend in general. Guo et al. (20) and Sun and Liu (21) found that the duration and cumulative temperature above key thresholds (≥0°C) in Northwest China increased consistently, at rates of 2.8d/10a and 72.8°C·d/10a, respectively and the increase was more obvious after 1995. Xu et al. (18) found that even though the increase in heat is consistent, the tendency is more obvious during the growing seasons of cool-loving crops relative to that of warm-loving crops, indicating the potential impacts on plant variety selection (22).

Water is a primary restricting factor for agricultural development in Northwest China (23). Climate change has led to changes in precipitation patterns, glacier and snow melting rates, as well as surface runoffs, which have changed the spatial and temporal distribution of water resources in Northwest China (24). Rise in precipitation and runoff are observed in the western part of Northwest China (18, 25–27), increasing the water resources available for agriculture (28, 29). However, the phenomenon is partly the result of increased glacial melting (30, 31), a process that is unlikely to sustain in the long term. Contrary to the western part, the water resources in the eastern part have shown a decreasing trend (18). Han et al. (25) also showed this regional difference and further indicated that the difference occurred mainly due to the increase in heavy precipitation in summer in the western part and the decrease in heavy precipitation in autumn in the eastern part. Despite the mixed patterns of changes, in general, Northwest China is still an arid and semi-arid region, and water resources are still extremely scarce.

CO_2_ is a major greenhouse gas and also affects crop yields through photosynthesis and other physiological processes. Crop model simulations generally predict that crop yields will benefit from the fertilization effects of increased CO_2_ concentration (32). For instance, a study by (32) projected that cereal yields in China in 2050 will increase by 13–22% due to CO_2_ increase and other climate effects, and the Northwest will have the most significant growth. However, the extent of CO_2_ fertilization effects remains controversial due to large uncertainties in model results (5, 33).

### Crop Phenology and Suitability

Agricultural practices in Northwest China are very diverse. The types of crops include food crops, cash crops, and some specialty crops, and the planting methods include spring sowing, summer sowing, irrigated agriculture, oasis agriculture, and rain-fed agriculture, etc. The crops with large planting areas include maize, cotton, and winter wheat. Studies have shown that climate change has shifted the sowing period of warm-temperature-loving crops such as maize and cotton earlier, with maize sowing 2 days earlier (34) and cotton sowing 5–12 days earlier (35). But winter wheat sowing is delayed by 2–3d/10a, and consequently the overwintering period is shortened by 5–6d/10a, and the full fertility period is shortened by 7–8d/10a (36). In general, the current climate change trend is favorable for warm-temperature-loving crops in the Northwest, but the safety threshold and long-term uncertainty still require further research.

Climate change has heterogeneous impacts on different crops and agricultural regions in the Northwest (37, 38). Higher temperature will increase yields of crops such as maize and cotton (39), but will reduce yields of crops such as potatoes and caraway (40, 41). For different agricultural regions, one study shows that climate change increases the yields of crops by 10–20% in irrigated oasis areas, while decreases the yields of crops by 10–20% in rain-fed dry cropping areas (42). The proportions of different crops are likely to change significantly due to future climate change.

### Pests and Weeds Control

Climate change has impacts on the spatial distribution and relative competitiveness of pests and weeds (43), meaning that the applications of pesticides and herbicides need to be significantly changed in Northwest China. First, climate change has broadened the habitats of pests, causing pests to expand to higher latitudes and altitudes. For example, the altitude at which wheat stripe rust (caused by Puccinia striiformis west) occurs in Longnan area has increased by about 100–300 m (42). Second, climate change prolongs the damage period of pests. The beginning date of wheat stripe rust occurrence in Longnan area from 1999 to 2005 was half a month earlier compared with 1991–1998, and the beginning date of wheat aphid occurrence in Wuwei City shifted nearly 1 month earlier from 1985 to 2003 (35). Third, climate change leads to accelerated growth and generational turnovers of pests, and increases the overall survival rates (44). Fourth, climate change have heterogeneous impacts on crops, pests, and natural enemies of pests, and the original ecosystem balance may be disrupted.

The impacts of climate change on weeds are similar to that on pests. Warmer temperatures extend weed distribution northward and to higher elevations, and higher CO_2_ concentrations usually enhance the relative competitiveness of weeds. Sun et al. (45) also suggested that increased CO_2_ concentration, increased ambient temperature and changes in precipitation could lead to more severe weed damage.

### Soil Fertility and Fertilizer Use

Climate change has significant impacts on soil fertility and fertilizer application (46). The quality of most cultivated land in Northwest China is fifth grade or below according to China's farmland quality standards, which are less fertile marginal land. Rising temperature accelerates the decomposition rate of soil organic matter, thus accelerating soil deterioration. Studies have shown that for every 1°C increase in temperature, soil organic carbon will be lost by 10% or more (47). The efficiency of fertilizers is also sensitive to changes in ambient temperature, especially for nitrogen fertilizers, since an increase in temperature will increase the release of fast-acting N in the soil and accelerate the release rate. Wang et al. (48) showed that for every 1°C increase in temperature, the amount of N released increased by 4% on average, and the release cycle was shortened by 3.6 days, thus the amount of fertilizer applied each time should be increased by about 4% to maintain the original fertilizer efficiency. In other words, climate change has in general increased the amount of fertilizer application in Northwest China.

### Extreme Climate Disasters Response

Droughts, extreme heat waves and frosts are the main climate-related hazards in Northwest China (49), and their frequency and intensity have changed due to climate change, requiring systematic strengthening of disaster response and mitigation strategies. First, the overall warming trend has increased the frequency of extreme droughts in this region, particularly during the growing seasons of major crops (50). He et al. (50) found that the wheat and maize planting areas that suffer from extreme droughts are expanding, and their Crop Water Deficit Index (CWDI) is growing, indicating more severe droughts.

The extreme heat waves in the Northwest have also showed an increasing trend, and the high temperatures that exceed the upper temperature limit for crop growth can significantly reduce crop yields. Ren and Yang (51) suggested that the number of days with extremely high temperatures in Northwest China increased from 1951 to 2000 at a rate of 0.5 days/40a.

In contrast to drought and extreme heat waves, extreme minimum temperatures and frost in Northwest China showed a decreasing trend. The first frost date was significantly delayed at an average rate of 1.8d/10a (52), while the last frost date was advanced at an average rate of 1.9d/10a in Northwest China from 1961 to 2009 (53). With longer frost-free periods and less frequent frosts, the damage caused by extreme minimum temperatures to agriculture was reduced (54). But Yao et al. (35) argued that due to its increased intensity, the low-temperature disaster damage could also increase.

## Strategies to Adapt to Climate Change

Section Impacts of Climate Change on Environmental Factors and Agricultural Practices in Northwest China reveals that climate change can have significant impacts on key environmental factors that agricultural production depends on, which can further affect agricultural practices in profound ways. Due to the uncertainty of climate change impacts, trends of environmental factors derived from different studies are not always consistent, and the impacts of climate change on agriculture practices in Northwest China are also mixed and uncertain. This section is intended to propose a series of potential adaptation strategies to cope with the long-term uncertain impacts of climate change.

### Optimizing Geographic Distribution of Agricultural Activities

Due to the geographic changes in heat and water resources as well as climate disasters, the spatial distribution of agricultural activities in Northwest China needs to make corresponding adjustments to better adapt to climate change (43). The bottom-line principle is that the majority parts of Northwest China are not suitable for further expansion of large-scale commercial agriculture, due to restrictions in environmental conditions, particularly water resources (55, 56). More specific spatial optimization of agriculture should be based on local conditions (57). First, in areas with favorable water resources, such as Guanzhong Plain, Hanzhong Plain, and Hetao Plain, agricultural infrastructure should be improved to sustain the existing irrigated farmlands and to improve water efficiency. Second, in rain-fed or oasis regions, it is important to develop agricultural systems with comparative advantages and regional characteristics, such as specialty crops that have high economic value and low water consumption rate (42). Fruits and nuts in Xinjiang are good examples for this type. Third, in ecologically vulnerable regions, such as the Loess Plateau that has suffered from frequent droughts and soil degradation, the agricultural areas should be reduced, and initiatives such as the “returning farmland to forest” and “returning farmland to grassland” programs should be promoted.

### Adjusting Crop Composition and Breeding New Varieties for Climate Adaptation

Adjusting the types of crops planted in different regions is needed in the changing environment (43, 58). For regions that rely primarily on surface river runoffs for irrigation, such as the Hetao Plain, planting of highly water-consuming crops, such as rice, should be restricted, considering the future uncertainty in water supply. In rain-fed agricultural areas, gradually reducing wheat production and increasing the production of more drought-resistant crops, such as potatoes, corn, and coarse cereals, will help better adapt to future climate change (58). In addition, selecting and breeding crop varieties with better adaptation ability is an important long-term strategy. Varieties selection and breeding should focus on two priorities. First, select and breed varieties with high resistance to drought, high temperature, and pests and diseases to increase the resilience to climate disasters (42, 47); Second, select and breed varieties with low water consumption rate and high photosynthetic productivity to reduce the environmental stress of agricultural systems and make use of the carbon fertilization effect.

### Enhancing Disaster Response Capacity of Agricultural Systems

Climate change will potentially intensify extreme climatic disasters in Northwest China, and it is necessary to improve the resilience of agricultural system at different levels. First, at the household level, farmers should be incentivized to adopt practices such as terraces, straw mulching, and sprinkler and drip irrigation, to improve their resilience to droughts and other disasters (47). Second, the government should establish agricultural disaster monitoring and warning systems to strengthen forecasting of extreme weather events (2), and should make emergency responding plans for different disasters. Third, agricultural safety networks, such as climate disaster insurances, should be built to mitigate the financial losses of individual farmers caused by extreme climate disasters (33). Fourth, it is important to maintain the integrity and stability of local agricultural systems and to strictly prevent the spread of invasive species, pests, and diseases caused by climate change (57).

### Strengthening Science and Technology Support for Climate Change Adaptation

Sustainable agriculture in the context of climate change needs extensive support from science and technology. First, research and development investment should be significantly increased in key fields, such as breeding, ecological-friendly fertilizers, pesticides, and herbicides, water-saving technologies, and other adaptation technologies. Innovative agricultural research should be promoted in research institutes, universities, and enterprises, and relevant departments, centers, and labs should be established and funded (57). Second, market incentive mechanisms for agricultural innovations should be established to facilitate the transferring of science and technology from labs to farmland. Intellectual properties should be protected, and market profits should be encouraged to attract more financial resources into sustainable agriculture (12). Third, strengthening the education and training of farmers, agricultural agency officials, and NGO members to spread agricultural science and technology knowledge to broader basis.

## Discussion

Mitigation and adaptation are two strategies to cope with climate change (59). Reducing agricultural greenhouse gases (GHG) emissions is essential, and mitigation measures, such as reducing fertilizer use and efficient use of agricultural waste, have also accomplished a certain amount of emissions reduction (60, 61). However, the share of GHG emissions from agriculture is small, and agricultural mitigation measures in Northwest China are far from sufficient to change the trend of climate. Therefore, adaptation should be the primary strategy in Northwest China' agricultural sector to take full advantage of the positive impacts of climate change and minimize the negative impacts (62–64).

**Figure 2:**
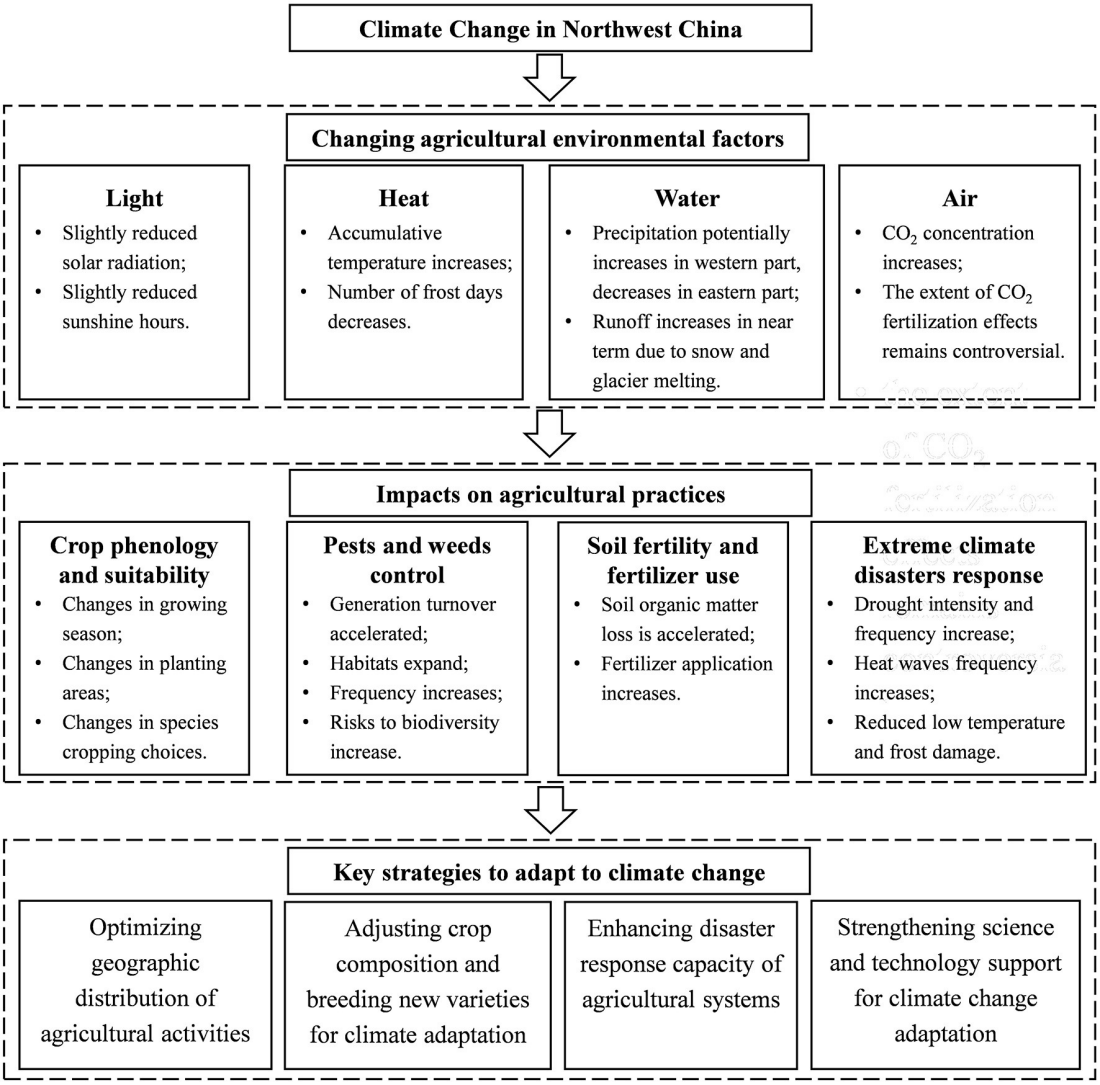
A framework for systematic thinking on sustainable agricultural development in Northwest China in the context of climate change.

This paper highlights the importance of putting climate change adaptation at the center of long-term policy making for sustainable agriculture development in Northwest China. We argue that it is important to have a systematic approach toward climate change adaptation, and propose a schematic framework for strategic thinking (Figure 2). The schematic framework establishes linkages among changing agricultural environmental factors, impacts on agricultural practices, and key adaptation strategies. Though it is not feasible to have an exhaustive review of literature in such a perspective article, our selected representative literature enables us to draw a big picture for the impacts of climate change and the responding strategies. This schematic framework is supposed to be used as a starting point for policy thinking, and each specific topic discussed in our paper requires more in-depth research and comprehensive understanding.

It is important to point out that the impacts of climate change on agriculture are mixed and uncertain. Some people may argue that climate change can be beneficial for certain agricultural regions, at least in the short term. However, if global mean temperature rise exceeds a safety threshold and triggers series of “tipping point” changes, the impacts on agriculture can be devastating. Scenario analyses show that even with adaptation strategies, future climate change will still cause a substantial loss in China's agriculture (63). Thus, for policy makers, it is important to “hope for the best, but prepare for the worst.” And for researchers, more in-depth studies are needed to better understand the safety threshold and long-term uncertainties of climate change, in order to better inform policy making.

## Data Availability Statement

The original contributions presented in the study are included in the article/supplementary material, further inquiries can be directed to the corresponding author/s.

## Author Contributions

DL, YL, and PuW contributed to conception and design of the study. DL and YL wrote the first draft of the manuscript. All authors contributed to the article and approved the submitted version.

## Funding

This study is funded by National Natural Science Foundation of China (NSFC) (Project No. 72004216).

## Conflict of Interest

The authors declare that the research was conducted in the absence of any commercial or financial relationships that could be construed as a potential conflict of interest.

## Publisher's Note

All claims expressed in this article are solely those of the authors and do not necessarily represent those of their affiliated organizations, or those of the publisher, the editors and the reviewers. Any product that may be evaluated in this article, or claim that may be made by its manufacturer, is not guaranteed or endorsed by the publisher.
